# Density of States for Warped Energy Bands

**DOI:** 10.1038/srep22098

**Published:** 2016-02-24

**Authors:** Nicholas A. Mecholsky, Lorenzo Resca, Ian L. Pegg, Marco Fornari

**Affiliations:** 1 The Catholic University of America, Department of Physics and Vitreous State Laboratory, Washington, DC 20064, USA; 2 Central Michigan University, Department of Physics and Science of Advanced Materials Program, Mount Pleasant, Michigan 48858, USA

## Abstract

Warping of energy bands can affect the density of states (DOS) in ways that can be large or subtle. Despite their potential for significant practical impacts on materials properties, these effects have not been rigorously demonstrated previously. Here we rectify this using an angular effective mass formalism that we have developed. To clarify the often confusing terminology in this field, “band warping” is precisely defined as pertaining to any multivariate energy function *E*(k) that does not admit a second-order differential at an isolated critical point in k-space, which we clearly distinguish from band non-parabolicity. We further describe band “corrugation” as a qualitative form of band warping that increasingly deviates from being twice differentiable at an isolated critical point. These features affect the density-of-states and other parameters ascribed to band warping in various ways. We demonstrate these effects, providing explicit calculations of DOS and their effective masses for warped energy dispersions originally derived by Kittel and others. Other physical and mathematical examples are provided to demonstrate fundamental distinctions that must be drawn between DOS contributions that originate from band warping and contributions that derive from band non-parabolicity. For some non-degenerate bands in thermoelectric materials, this may have profound consequences of practical interest.

The density of states (DOS) in electronic energy space, usually denoted as *g*(*E*), is a fundamental quantity in solid state physics, which critically determines transport, optical, and many other properties of materials^1^,^2^,^3^,^4^,^5^. In fact, *g*(*E*) is most immediately responsible for those properties, and more directly related to their corresponding measurements than the underlying band structure that generates *g*(*E*). Materials that have quite similar densities of states typically display quite similar properties, even though their underlying band structures may differ.

Effective mass approximations of energy dispersions are central in analyzing and understanding band structures of materials near critical points in the Brillouin zone (BZ) and their major physical consequences on DOS^6^. However, basic formulae of that formalism have been misused for energy band dispersions that are not twice-differentiable at isolated critical points in the BZ. That is what is generally, and should be exclusively, called “band warping.” Unfortunately, at times band warping has been further confused with band non-parabolicity for energy functions *E*(**k**) that do admit Taylor series expansions at critical points in **k**-space. In a previous paper, a mathematically and physically rigorous theory for treating a broad class of energy band dispersions in crystals has been introduced to correct these matters^7^. That formalism, which is based on angular effective mass expansions^7^, can be used to provide rigorous expressions and reliable calculations of densities of states originating from any underlying band structure. Surprisingly, that has not been done for warped energy bands heretofore. Thus, the main purpose of this paper is to remedy this major deficiency in energy band structure theory.

Let us begin by recalling the radial expansion of an energy band around a point **k**_0_ in a crystal BZ, expressed parametrically in angular form as^7^


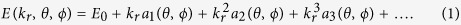


In Eq. (1), 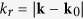 is the radial distance between a generic point at **k** in the BZ and **k**_0_. This **k**_0_ may be any point of special interest in the BZ, or a “critical point,” meaning that the energy expansion has a zero first-order differential at **k**_0_^1^,^2^. The polar angles *θ* and *ϕ* refer to the spherical polar coordinates of 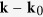.

It is essential to appreciate that Eq. (1) applies to far more general dispersion relations than commonly considered multi-dimensional Taylor series expansions in Cartesian coordinates. That is so because Eq. (1) requires only the existence of a one-dimensional Taylor series expansion in each radial direction through **k**_0_. This is a much more limited requirement and is reasonably expected of any physical band structure that allows one-dimensional transport of quasi-particles in any direction^7^. Besides ordinary quadratic bands, Eq. (1) thus includes “warped bands,” which are *not* twice-differentiable at isolated points, based on a rigorous mathematical definition. Typical examples of warped bands derive from original models of Dresselhaus *et al.* and Kane^8^,^9^.

Both physically and mathematically, band warping must be unambiguously distinguished from band non-parabolicity. The latter derives from higher-order terms 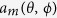 with 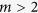 in Eq. (1). Conversely, band warping depends exclusively on the shape of the 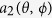 term, which provides a dimensionless angular effective mass surface in Rydberg atomic units^7^.

For an analytically quadratic band, associated with a proper second-order differential, its curvature, 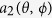, must assume the form


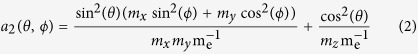


in a coordinate system of principal axes, with diagonal effective masses 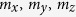. In Eq. (2), m_e_ is the ordinary electron mass. Any other form of 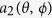 that cannot be exclusively described in terms of diagonal effective masses and orthogonal principal axes must correspond to a “warped band.”

One may formally derive expressions for the DOS corresponding to the general energy expansion in Eq. (1). In this paper, we focus on explicit DOS expressions for band warping, although we generalize our considerations at least to one type of band non-parabolicity, namely that of an overall energy dispersion of the form 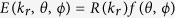, where *R*(*K_r_*) is a monotonically increasing function, implying that all 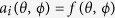 coincide. We do not further consider in this paper any linear term in the energy expansion, Eq. (1), thus assuming a zero first-order differential at a “critical point.”^1^,^2^,^7^

We begin our technical presentation by deriving a general expression for the DOS and the DOS effective mass for any physical energy dispersion in two- and three-dimensional reciprocal spaces. We verify that our expression correctly reproduces standard results for the DOS of non-warped, i.e., at least twice differentiable, energy dispersions. We then proceed to demonstrate the effect of band warping on the DOS by applying our expression to a fundamentally warped energy dispersion originally derived by Dresselhaus, Kip and Kittel^8^. We show that there are considerable differences between our correct evaluations of DOS effective masses and those erroneously produced in original papers^10^,^11^,^12^ and reproduced ever since. In the Supplemental Material to this paper, we further discuss two-dimensional mathematical models, where the distinction between effects of band warping – and a particular form of it that may be associated with the idea of “corrugation” – and effects of band non-parabolicity can be analytically demonstrated. Some features of those examples can be critical to clarify the interplay between the possibilities of band warping and band non-parabolicity in non-degenerate bands of materials that exhibit remarkable thermoelectric properties in that connection^13^,^14^,^15^,^16^,^17^,^18^,^19^.

## Results

The DOS for a warped energy dispersion must be obtained using our angular formalism. We thus proceed to derive its appropriate expression, first in three dimensions and then in two dimensions.

### Density of States Formulae

In a crystal, the single-band DOS at energy *E*, within *dE*, is defined as


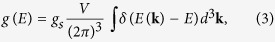


where *g_s_* is a possible spin degeneracy, *V* is the volume of the direct-lattice primitive cell, and *E*(**k**) represents a single energy band in the BZ over which the *d*^3^**k** integration is performed. A general strategy is to evaluate the integral by performing a transformation to (*E*, *θ*, *ϕ*) coordinates. The delta function can further be handled by reducing the integral to the surface having given energy *E* inside the BZ^1^,^2^,^3^.

Using this Eq. (3), we use a general dispersion given in angular form to derive an expression for the DOS.

### The DOS of Warped and Non-Warped Bands

Approaching a critical point **k**_0_ in the BZ, let us ignore band non-parabolicity effects for the moment and consider an energy dispersion (without any linear term) in the form^7^


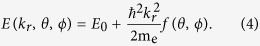


In Eq. (4) we imply a partition of the unit (*θ*, *ϕ*) sphere *S*^2^ in a region 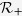 where 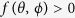 and a region 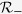 where 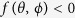, so that 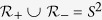. This definition of 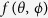 must refer to a single band, which may or may not be degenerate with other bands at **k**_0_. Typically, though not necessarily^13^,^14^,^15^, non-degenerate bands at **k**_0_ are not warped, corresponding to analytic maxima, minima, or saddle points. Conversely, degenerate bands are commonly warped^7^,^8^,^9^.

With the change of variables to 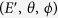, the DOS integral becomes


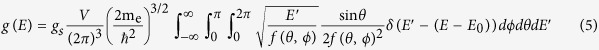


where *E*′ is an integration variable comprising the energy difference 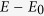 from the extremum of energy *E*_0_. The delta function then reduces integration to a surface integral over a surface of constant 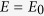.

In order to confirm the correctness of this expression, we may first reproduce standard results for the DOS of non-warped energy dispersions, and then proceed to evaluate the DOS for warped energy dispersions.

### DOS for Spherical, Ellipsoidal, Saddle, or Warped Dispersions

If angular integrations over the unit sphere converge, we may split those integrals over regions of positive and negative 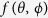, so that the energy integration immediately yields


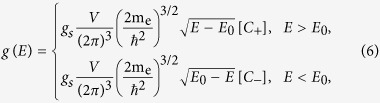


where we have defined


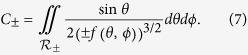


However, integration over the angular variables may not formally converge, as in the classic case of a saddle point dispersion extending to infinity^1^. That is a theoretical extrapolation, however, because the BZ is actually finite, and so must be any band structure within it. Introduction of an energy-dependent cutoff parameter may thus be required, which should further take into account the onset of any significant band non-parabolicity. In any such case, the energy integration must be taken last, since *C*_±_ also become functions of energy. However, the presence of the delta function can still make this last integration over energy relatively straightforward. We provide an example of that in the Supplemental Material.

### Generalization to Monotonically Non-Parabolic Bands

We can readily extend the preceding formalism to energy dispersions of the form 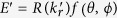 where 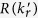 is any monotonically increasing function of 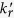, yielding the inverse function 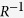.

The DOS thus becomes


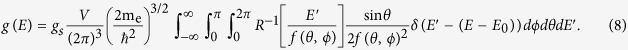


Equation (5) may now be regarded as a special case of Eq. (8), where 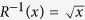. The energy integral may still be relatively straightforward to perform in Eq. (8) on account of the delta function.

For the sake of clarity and completeness, let us also derive corresponding expressions for the DOS in two dimensions.

### Two-dimensional DOS

Let us consider the two-dimensional evaluation of the DOS, according to the expression


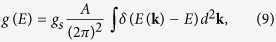


where *g_s_* is the spin degeneracy and *A* is the area of the direct-lattice primitive cell. Close to a critical point **k**_0_ in the BZ, and ignoring band non-parabolicity, the two-dimensional energy dispersion becomes


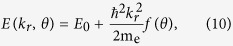


where *f*(*θ*) is now a function of a single angular variable. The Jacobian of the transformation from rectangular to polar coordinates is simply


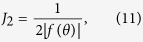


and the DOS thus becomes


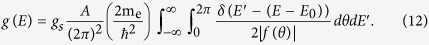


Again, we must integrate over regions of positive and negative *f*(*θ*) separately. Namely, the interval (0, 2*π*) must be split into 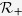 and 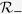 regions, where *f*(*θ*) is either positive or negative, respectively. Assuming that corresponding *θ*-integrals converge, this yields


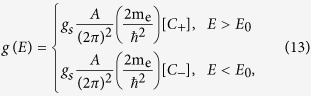


where


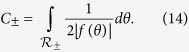


Using this formula, Eq. (14), and its three dimensional counterpart, Eq. (7), we may now precisely define the DOS effective mass from comparison with the spherical dispersion case.

### The DOS Effective Mass

Since the form of Eq. (4) is devised to further capture band warping at a critical point in the BZ, we may use the standard expression for the DOS effective mass in the spherical case


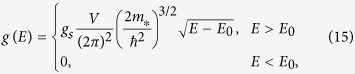


to define the DOS effective mass for a warped energy band minimum, or its straightforward modification for an energy band maximum. Comparing Eq. (6) with Eq. (15), and recalling the definitions of the numerical factors given in Eq. (7), we may generally define the DOS effective mass as


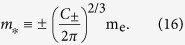


In the two-dimensional case, we can similarly introduce


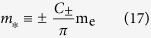


in Eq. (13).

### The DOS Effective Masses for the Kittel Form

#### Three-dimensional Kittel form

As a basic illustration of our results, let us calculate the DOS effective masses for the hole bands described by what we may dub the “Kittel form,” originally derived in a ground-breaking paper^8^, as


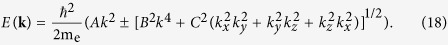


Expressing that according to our Eq. (4), we obtain exactly^7^


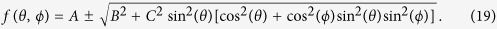


In both expressions, the upper positive (lower negative) sign refers to the heavy (light) hole band dispersion, and *A* < 0.

Although we may not be able to express it in a closed analytic form, each DOS effective mass for the Kittel form can be evaluated numerically using Eq. (7) and Eq. (16). Let us further factorize the absolute value of the *B* parameter in front of the energy dispersion of the Kittel form or its angular effective mass surface.

**Figure 1:**
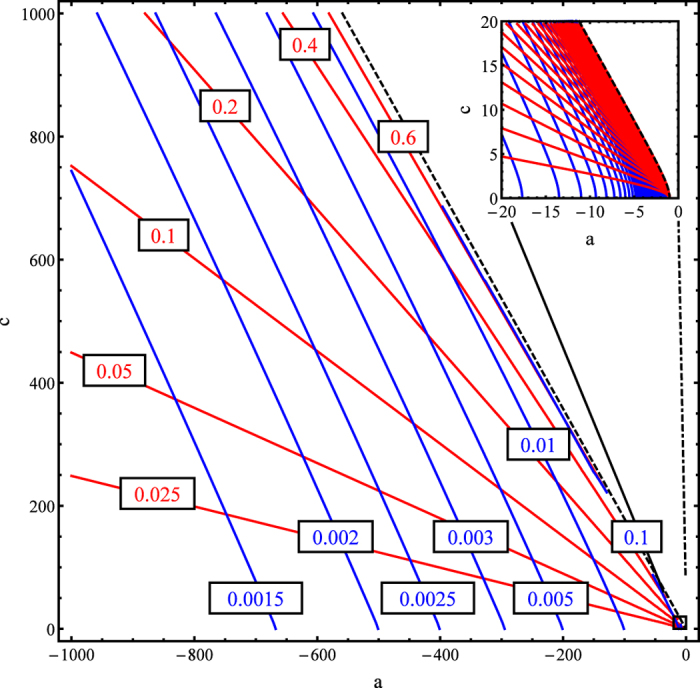
Contour plots of constant DOS heavy-hole effective mass *m*_hh_, in units of *m*_e_|*B*, are shown as blue curves. For comparison, contour plots of constant absolute value of warping parameter *w* are shown as red curves.

In Fig. 1 we have numerically calculated the heavy-hole DOS effective mass for a large region of the parameter space of possible *A*, *B* and *C* values in the Kittel form. Furthermore, contours of the corresponding DOS heavy-hole effective mass, *m*_hh_, are shown in blue in Fig. 1 as functions of 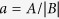 and 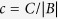. Numerical values of *m*_hh_ are given in units of m_e_|*B*.

Notice that 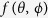 becomes imaginary for values of *θ* and *ϕ* if *c* exceeds a *c*_max_ given by


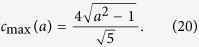


Contours of constant *m*_hh_ thus appear to accumulate along a corresponding curve. It is not apparent whether any *m*_hh_ may be attained for values of *a* and *c* approaching Eq. (20) from below. For example, the parameters for the valence bands of Si are given in Ref. 7 as 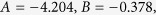 and 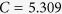. This corresponds to a (hh) DOS effective mass of 0.592 m_e_ and a (lh) DOS effective mass of 0.155 m_e_.

We may also compute the band warping parameter, *w*, previously defined in Ref. 7, for the heavy-hole band of the Kittel form. This warping parameter gives some measure of how distorted from a quadratic Taylor expansion the energy band structure is around a critical point. Contour plots of constant *w* are shown in red in Fig. 1. Notice that, moving along curves of constant *w*, the DOS heavy-hole effective mass *m*_hh_ increases with increasing *a*. Alternatively, moving along curves of constant *m*_hh_, the band warping parameter *w* increases with increasing *c*. Thus, perhaps surprisingly, a larger value of *w* does not necessarily imply either a larger or a smaller value of *m*_hh_, since that depends on the values of *a* and *c* parameters; and conversely.

#### Two-dimensional Kittel form

We may reduce the previous results to a two-dimensional version of the Kittel form determined by setting 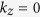 in Eq. (18), namely,


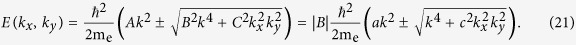


Equivalently, by setting 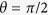 in Eq. (19), and then relabeling the azimuthal angle *ϕ* with the two-dimensional polar angle *θ*, we obtain exactly


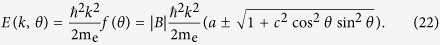


**Figure 2:**
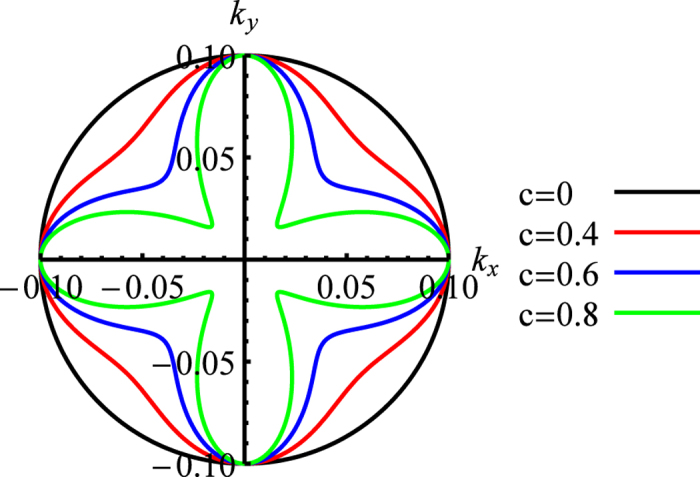
Angular effective mass contours of *f*(*θ*) for the two-dimensional Kittel form that has *k_z_* = 0, for parameter values of *a* = -1.1 and *c* = 0.0, 0.4, 0.6, and 0.8. The dependence is exactly parabolic in every radial direction.

Angular effective mass planar contours of *f*(_θ_) are shown in Fig. 2 for a given value of *a* and four increasing values of the *c* parameter. In this two-dimensional case, the band warping parameter, *w*, and the DOS effective mass, *m*_∗_, can be expressed analytically, for any 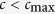, as


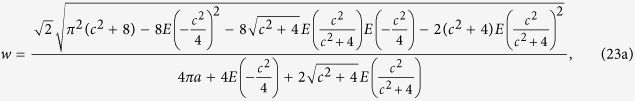



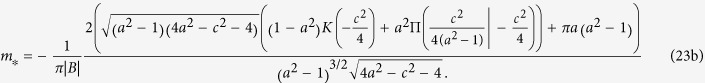


In Eq. (23), *E*(*m*), *K*(*m*), and 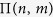 denote the complete elliptic integral, the complete elliptic integral of the first kind, and the complete elliptic integral of the third kind, respectively, and 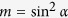 and *n* are their standard arguments.

Contours of constant DOS heavy-hole effective mass *m*_hh_ and contours of constant absolute value of warping parameter, *w*, for this two-dimensional Kittel form are qualitatively similar to those of the full three-dimensional Kittel form (c.f. Fig. 1). However, in the two-dimensional case, the DOS effective mass decreases away from a maximum limiting value at *c* = 0 and *a* = -1. In two dimensions *w* attains a maximum magnitude 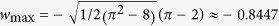 whenever *a* and *c* approach the limit of *c*_max_. Additionally, the expression for the effective mass in Eq. (23) has a limiting value at *c* = 0 and 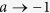 from the left. This limiting value for the two-dimensional case is 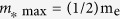. We did not investigate a corresponding effect for the full three dimensional Kittel form. We expect a similar result for the warping parameter, but it is uncertain whether or not there may be a theoretical maximum value for the DOS effective mass.

## Discussion

Our formulae reproduce expected results for ellipsoidal and hyperboloidal quadratic energy expansions. For the latter, we arrive at a result similar to that of saddle points in Ref. 1.

To further extend our results to optical transitions, the joint density of states (JDOS) can be similarly considered^1^,^2^. Both conduction and valence bands can be expressed as individual terms having the form of Eq. (4). For the JDOS we may then define a joint *F*(*θ*, *ϕ*) as the sum of the corresponding two (absorbing and emitting) *f*-contributions. The same formalism that we develop in this paper for the DOS thus essentially applies to the JDOS as well.

Given our somewhat unexpected results concerning independence of DOS from band warping and structure in the Kittel form, it is natural to question what effects or relations may generally exist between band warping and DOS effective masses. In any case, if we consider energy dispersions with angular contributions giving rise to finite *C*_±_ in Eq. (7), then the only effect that band warping can have on the DOS is to modify the numerical factor in Eq. (6).

In the Supplemental Material, we investigate several models that clearly show that band warping may or may not increase the DOS effective mass. Additionally, an intuitive notion of greater band “corrugation,” referring to energy dispersions that deviate “more severely” from being twice-differentiable at an isolated critical point, may also vary independently of the corresponding DOS effective mass *and* the band warping parameter. For example, in addition to constructed example dispersions where warping is independent of the DOS effective mass, we provide examples where the warping parameter steadily increases with what we dub band “corrugation,” whereas the DOS effective mass at first decreases, but then increases with that “corrugation.”

**Figure 3:**
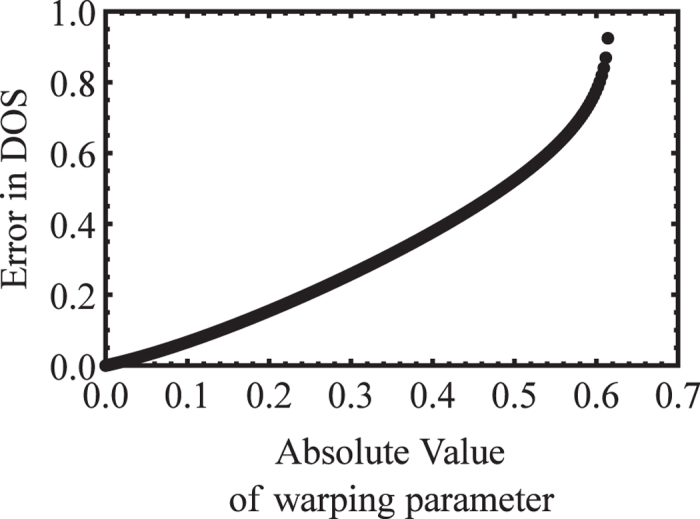
Relative error versus band warping parameter, 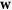, for the Kittel form. For specificity, we start at a point 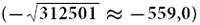 in Fig. 1, and then we increase 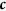 vertically. Evidently, the relative error of the DOS effective mass, derived from the least-squares fit of the angular effective mass surface to a spherical surface, increases monotonically with 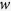.

For the Kittel form, we find that the warping parameter, *w*, may be used to indicate how far from spherical is the angular effective mass surface 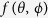. For example, in the plane (*a*, *c*)of Fig. 1, if we climb vertically along the positive *c* axis from some point, e.g. 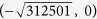, *w* increases. The particular 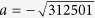 value has been chosen simply to let *c* range from 0 to 1000. Let us then compute the error between an approximate DOS effective mass, derived from the least-squares fit of the 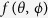 surface to a sphere and then using the effective mass formula Eq. (15), and the correct DOS effective mass, calculated from Eq. (7). That error is plotted in Fig. 3. As expected, when *c* = 0, the relative error 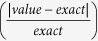 is zero, because the angular effective mass surface is actually spherical. However, as *c* (and correspondingly *w*) increase, the relative error 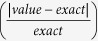 increases up to almost 100%! This highlights the need for the correct DOS effective mass formula, rather than approximating a warped surface with an ellipsoid, as is typically done incorrectly.

Lax and Mavroides^10^ originally proposed the correct idea of an angular effective mass, but they immediately deviated from it to fit the Kittel form specifically. Their Eq. (8) and those at the beginning of their Sec. IIIA correspond to our Eqns. (7) and (16), in defining the DOS effective mass. However, not only is our treatment much more general than theirs, but it also applies more appropriately to the Kittel form, based on Eq. (19). Additionally, the DOS effective mass formula that we derive is similar to the CC mass developed in Ref. 20 for Si and Ge, although those authors refer to ‘nonspherical-nonparabolic’ band structures, whereas we more precisely consider parabolic, although possibly warped, band structures.

Our treatment of the DOS effective masses can also be contrasted with that of Lawaetz^12^. Using our correct expressions and integrating them numerically for the same values of parameters reported by Lawaetz for various materials, there are significant differences between our appropriate DOS effective masses and those artificially produced by Lawaetz’s formulae. We show that in Table 1, where we have used the following relations between the *A*, *B*, and *C* parameters of the Kittel form and the *γ*_1_, *γ*_2_ and *γ*_3_ parameters introduced by Luttinger^21^,


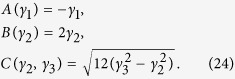


**Figure 4:**
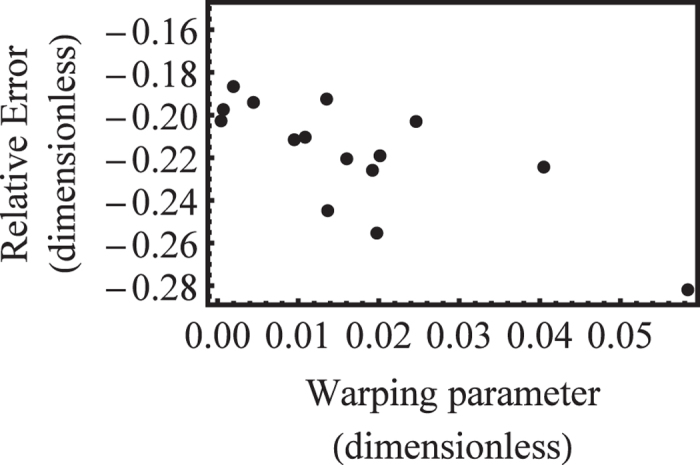
Relative error of the DOS heavy-hole effective masses 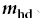 estimated by Lawaetz^12^ and reported in column 5 of Table 1, as compared to our correct values, computed from Eq. (7) and Eq. (16) and reported in column 6 of Table 1, versus the band warping parameter 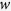.

Figure 4 shows the error of the DOS heavy-hole effective mass estimated by Lawaetz and its correlation with the warping parameter *w* for that band in various materials. That error is partly the result of inconsistent series expansions and truncations in procedures elaborated by Lax, Mavroides and Lawaetz^10^,^11^,^12^. Roughly, the larger the warping, or *w*, the greater is the discrepancy between Lawaetz’s estimate and our precise determination of the DOS effective mass, consistent with the discussion of the Kittel form above. That error can be quantitatively as large as 28% depending on the material. More importantly, the original lack of a precise definition and treatment of warped bands has been responsible for a lack of consistency among many subsequent papers and *ad hoc* estimates of the DOS effective masses.

## Methods

### Derivation of Density of States formulae

To proceed with the integrations in Eq. (3), we scale the Cartesian coordinates by letting 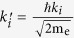. The energy dispersion thus becomes


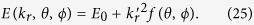


We may then introduce a new variable 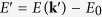, so that


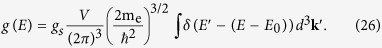


In polar coordinates we have


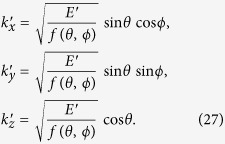


Accordingly, we may perform a change of variables to spherical coordinates (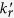, *θ*, *ϕ*), where 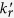 is defined implicitly through Eq. (25). Notice that regions of positive *E*′(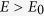) correspond to 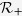 and regions of negative *E*′ (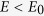) correspond to 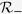, so that all variables in Eq. (27) are real.

The Jacobian for this transformation is


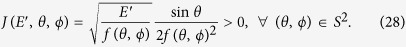


The corresponding coordinate transformations for the generalized formulae of monotonically increasing non-parabolic bands then become


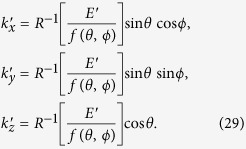


The inverse function *R*^-1^ of *R* introduced in Eq. (29) yields Eq. (8).

## Additional Information

**How to cite this article**: Mecholsky, N. A. *et al.* Density of States for Warped Energy Bands. *Sci. Rep.* **6**, 22098; doi: 10.1038/srep22098 (2016).

## Supplementary Material

Supplementary Information

## Figures and Tables

**Table 1:** Comparison of the DOS effective masses for materials reported in Table II of ref. 12. and those correctly derived from our Equations (7) and (16).

Crystal	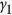	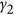	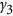	Lawaetz 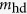	Correct 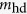	Lawaetz 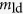	Correct 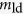
C	4.62	−0.38	1.00	^*a*^	^*a*^	^*a*^	^*a*^
Si	4.22	0.39	1.44	0.53	0.537	0.16	0.156
Ge	13.35	4.25	5.69	0.35	0.351	0.043	0.0423
Sn	−14.97	−10.61	−8.52	0.29	0.289	−0.029	−0.0297
AlP	3.47	0.06	1.15	0.63	0.615	0.2	0.195
AlAs	4.04	0.78	1.57	0.76	0.752	0.15	0.151
AlSb	4.15	1.01	1.75	0.94	0.953	0.14	0.141
GaP	4.2	0.98	1.66	0.79	0.786	0.14	0.143
GaAs	7.65	2.41	3.28	0.62	0.620	0.074	0.0739
GaSb	11.8	4.03	5.26	0.49	0.498	0.046	0.0468
InP	6.28	2.08	2.76	0.85	0.858	0.089	0.0887
InAs	19.67	8.37	9.29	0.60	0.600	0.027	0.0267
InSb	35.08	15.64	16.91	0.47	0.490	0.015	0.0147
ZnS	2.54	0.75	1.09	1.76	1.796	0.23	0.224
ZnSe	3.77	1.24	1.67	1.44	1.468	0.149	0.148
ZnTe	3.74	1.07	1.64	1.27	1.296	0.154	0.152
CdTe	5.29	1.89	2.46	1.38	1.466	0.103	0.102
HgS	−41.28	−21	−20.73	2.78	2.946	−0.012	−0.0121
HgSe	−25.96	−13.69	−13.2	1.36	1.341	−0.019	−0.0190
HgTe	−18.68	−10.19	−9.56	1.12	1.220	−0.026	−0.0261

^*a*^Formalism invalid because *γ*_2_ and *γ*_3_ have opposite sign.
