# Supplementary material for: Density of States for Warped Energy Bands
Source: Sci Rep. 2016 Feb 24;6:22098. doi: 10.1038/srep22098 (PMC4764916; doi:10.1038/srep22098)
Supplement: Supplementary Information [file srep22098-s1.pdf]

Supplemental Material
for
Density of States for Warped Energy Bands

Nicholas A. Mecholsky[1,*], Lorenzo Resca[1], Ian L. Pegg[1], and Marco Fornari[2]

[1]The Catholic University of America, Department of Physics and Vitreous State Laboratory, Washington, DC 20064, USA
[2]Central Michigan University, Department of Physics and Science of Advanced Materials Program, Mount Pleasant, Michigan 48858, USA
[*]nmech@vsl.cua.edu

Density of States in Ellipsoidal and Hyperbolic Dispersions

Ellipsoidal Energy Dispersions

As a first check, let us consider the basic case of an ellipsoidal energy dispersion of the form

$$E(k_x,k_y,k_z) = \frac{\hbar^2}{2m_x}k_x^2 + \frac{\hbar^2}{2m_y}k_y^2 + \frac{\hbar^2}{2m_z}k_z^2 + E_0, \tag{1}$$

where all three principal masses m_x, m_y, and m_z have the same sign. In a four-dimensional space where $E(k_x,k_y,k_z)$ refers to the fourth dimension, that provides a paraboloid with a minimum (maximum) if all three masses are positive (negative). For positive masses, we may rescale the corresponding coordinates as $k_i' = \frac{\hbar k_i}{\sqrt{2m_i}}$. The DOS is then expressed as

$$g(E) = g_s \frac{V}{(2\pi)^3} \frac{2^{3/2}\left(m_x m_y m_z\right)^{1/2}}{\hbar^3} \int \delta\left(E' - (E - E_0)\right) d^3\mathbf{k}', \tag{2}$$

where $E' = k_r'^2$, and $f(\theta,\phi) = 1$. Integration is straightforward, yielding

$$C_+ = 2\pi, \quad C_- = 0. \tag{3}$$

We thus recover the standard result

$$g(E) = \begin{cases} g_s \frac{V}{(2\pi)^2} \frac{2^{3/2}\left(m_x m_y m_z\right)^{1/2}}{\hbar^3} \sqrt{E - E_0}, & E > E_0, \\ 0, & E < E_0. \end{cases} \tag{4}$$

In the special case where all masses equal a single effective mass, $m_x = m_y = m_z = m_*$, corresponding to a spherical energy dispersion, we arrive at Eq. (15) in the main paper.[1]

Comparing Eqns. (4) and (15) in the main paper,[1] the usual DOS effective mass, $m_* = \left(m_x m_y m_z\right)^{1/3}$ for an ellipsoidal energy dispersion, is obtained.

If all three masses are initially negative, essentially equivalent results can be obtained for the DOS by switching the signs of all three masses to positive, while correspondingly switching the signs of all energies to negative in the preceding equations, starting with Eq. (1).

Hyperbolic Energy Dispersions

A hyperbolic energy dispersion still has the form of Eq. (1), but some of the masses have opposite signs. For the sake of simplicity, although without any major loss of generality, let us posit that $m_x = m_y = -m_z = m_e$ in Eq. (1). In that case, the

angular effective mass surface $f(\theta,\phi)$ in Eq. (2) and Eq. (4), and its corresponding $\mathscr{R}_\pm$ regions, are

$$f(\theta,\phi) = -\cos(2\theta),$$
$$\mathscr{R}_+ = \left\{(\theta,\phi)\,\middle|\,\frac{\pi}{4} < \theta < \frac{3\pi}{4}\right\}, \tag{5}$$
$$\mathscr{R}_- = \left\{(\theta,\phi)\,\middle|\,0 < \theta < \frac{\pi}{4}\text{ or }\frac{3\pi}{4} < \theta < \pi\right\}. \tag{6}$$

As expected for an endless hyperbolic dispersion, the surface integrals do not converge and a 'spherical' cutoff radius R_c must be introduced.[2] In turn, this introduces an energy-angle relation at R_c, namely $\pm E' = -R_c^2\cos 2(\frac{\pi}{4}\pm\varepsilon)$, where ε represents the angular increment from $\frac{\pi}{4}$ corresponding to the intersection of the sphere of radius R_c and the hyperboloid of constant E'. Now the surface integrals based on Eq. (7) can be formally performed, yielding

$$\begin{aligned} C_+(E';R_c) &= 2\cdot 2\pi\int_{\frac{\pi}{4}+\varepsilon}^{\frac{\pi}{2}} \frac{\sin\theta}{2(-\cos 2\theta)^{3/2}}\,d\theta \\ &= \frac{-\cos\theta}{2\sqrt{-\cos 2\theta}}\Bigg|_{\frac{1}{2}\cos^{-1}\left(\frac{E'}{R_c^2}\right)}^{\frac{\pi}{2}} = \frac{2\pi}{\sqrt{2}}\frac{R_c}{\sqrt{E'}} + \frac{\pi}{\sqrt{2}}\frac{\sqrt{E'}}{R_c} - \frac{\pi}{4\sqrt{2}}\frac{(E')^{3/2}}{R_c^2} + \ldots \end{aligned} \tag{7a}$$

$$\begin{aligned} C_-(E';R_c) &= 2\cdot 2\pi\int_0^{\frac{\pi}{4}-\varepsilon} \frac{\sin\theta}{2(\cos 2\theta)^{3/2}}\,d\theta \\ &= \frac{\cos\theta}{2\sqrt{\cos 2\theta}}\Bigg|_0^{\frac{1}{2}\cos^{-1}\left(\frac{-E'}{R_c^2}\right)} = \frac{2\pi}{\sqrt{2}}\frac{R_c}{\sqrt{-E'}} - 2\pi + \frac{\pi}{\sqrt{2}}\frac{\sqrt{-E'}}{R_c} - \frac{\pi}{4\sqrt{2}}\frac{(-E')^{3/2}}{R_c^2} + \ldots. \end{aligned} \tag{7b}$$

We may thus perform, in Eq. (5) of the main paper[1], the energy-dependent angular surface integrals over θ and ϕ of $C_\pm$ and complete the energy integration via the delta function, thus obtaining

$$g(E) = \begin{cases} g_s\frac{V}{(2\pi)^3}\left(\frac{2\mathrm{m_e}}{\hbar^2}\right)^{3/2}\left[\frac{2\pi}{\sqrt{2}}R_c + \frac{\pi}{\sqrt{2}}\frac{E-E_0}{R_c} - \frac{\pi}{4\sqrt{2}}\frac{(E-E_0)^2}{R_c^2} + \ldots\right], & E > E_0, \\ g_s\frac{V}{(2\pi)^3}\left(\frac{2\mathrm{m_e}}{\hbar^2}\right)^{3/2}\left[\frac{2\pi}{\sqrt{2}}R_c - 2\pi\sqrt{E_0-E} + \frac{\pi}{\sqrt{2}}\frac{E_0-E}{R_c} - \frac{\pi}{4\sqrt{2}}\frac{(E_0-E)^2}{R_c^2} + \ldots\right], & E < E_0. \end{cases} \tag{8}$$

This agrees with the form of the DOS around an M_1 saddle point, as derived on p. 157 of Ref. [2], for example.

A Band Warping Parameter

There are multiple ways of introducing parameters that provide some measures of band warping. Conversely, no single parameter can be expected to account entirely for the full angular complexity of $f(\theta,\phi)$. We have previously introduced one measure of band warping by defining a parameter[3]

$$w = \frac{\langle\,(Tr[H] - \langle Tr[H]\rangle)^2\,\rangle^{1/2}}{\langle Tr[H]\rangle}. \tag{9}$$

For the sake of illustration, let us return to a two-dimensional energy dispersion as in Eq. (10) of the main paper[1], namely, $E = \frac{\hbar^2k^2}{2\mathrm{m_e}}f(\theta)$, and let us further assume that $f(\theta)$ is positive everywhere. Then we have[3]

$$\langle\cdot\rangle_\theta = \frac{1}{2\pi}\int_0^{2\pi}\cdot\,d\theta,$$
$$Tr[H] = 2f(\theta) + 2f(\theta+\frac{\pi}{2}). \tag{10}$$

This definition of the band warping parameter, w, essentially measures the coefficient of variation of the sum of the eigenvalues of the Hessian matrix $H(\theta)$ formally obtained in each Cartesian coordinate system rotated by an angle θ. A twice-differentiable, i.e., a non-warped surface cannot have any variation of its quadratic form eigenvalues. Hence, that must have $w = 0$. However, the converse is not necessarily true. Namely, having $w = 0$ is not sufficient to conclude that the energy dispersion is twice-differentiable. The same considerations also apply to $f(\theta)$ curves with positive and negative values in different regions of S.

Two-Dimensional Examples of Energy Dispersions

Example of $a(1+b\cos 4\theta)$)

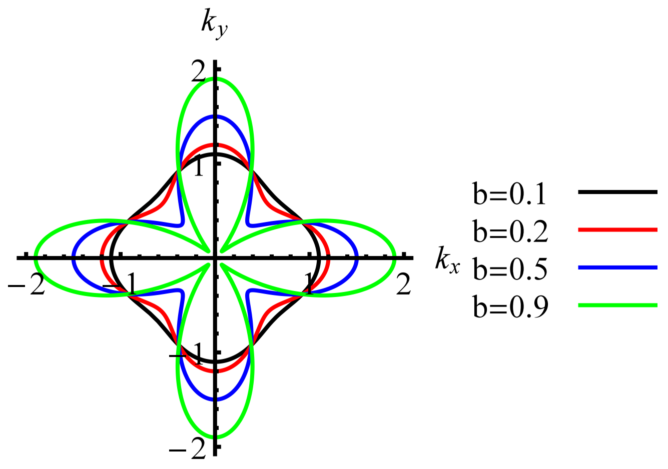

Figure 1. Angular effective mass contours of $f(\theta)$ for a two-dimensional dispersion relation of the form $E = \frac{\hbar^2 k^2}{2\mathrm{m_e}}(a+b\cos 4\theta)$, where we set $a = 1$, and $b = 0.1, 0.2, 0.5$, and 0.9.

In the main paper[1], we considered a two-dimensional example of the Kittel form. As a second example, we now consider the two-dimensional energy dispersion

$$E = \frac{\hbar^2 k^2}{2\mathrm{m_e}} f(\theta) = \frac{\hbar^2 k^2}{2\mathrm{m_e}} a(1+b\cos 4\theta). \tag{11}$$

Unless $b = 0$, this function is not twice-differentiable at the origin exclusively, as an isolated point. In Fig. 1, its angular effective mass $f(\theta)$ is plotted for $a = 1$ and four increasing values of b. Since the integrand in Eq. (14) of the main paper[1] consists of $\frac{1}{2|f(\theta)|}$, we can indefinitely decrease m_* in Eq. (16) by letting a become as small as we wish. On the other hand, for any given value of a, we expect that the main contributions to m_* derive from nearly diagonal directions, along which $|f(\theta)|$ becomes increasingly smaller as b approaches 1 from below. Consistently with those and other expectations, analytic derivations provide

$$w = \frac{b}{\sqrt{2}}, \tag{12}$$

$$m_* = \frac{1}{a\sqrt{1-b^2}}, \tag{13}$$

where $m_* = C_+/\pi$. Thus, interestingly, the band warping parameter, w, and the DOS effective mass, m_*, are independent of each other, since only m_* depends on a. Complete independence between w and m_* may have not been anticipated.

Example of $n^2\left(\cos^{2n} n\theta + \sin^{2n} n\theta\right)$

Let us now provide a more complex example where w steadily increases with what we may dub band "corrugation," whereas m_* at first decreases, but then increases with that "corrugation." Consider an energy dispersion of the form

$$E = \frac{\hbar^2 k^2}{2\mathrm{m_e}} f(\theta) = \frac{\hbar^2 k^2}{2\mathrm{m_e}} \left(n^2\left(\cos^{2n} n\theta + \sin^{2n} n\theta\right)\right). \tag{14}$$

Again, unless $n = 1$, this function is not twice-differentiable at the origin exclusively, as an isolated point. In Fig. 2 we show plots of its angular effective mass $f(\theta)$ for $n = 1$, 2, 3, and 4.

The impression conveyed by Fig. 2 is that the energy dispersion ought to deviate more and more from being twice-differentiable with increasing n. We are thus led to regard n as an alternative indicator of band warping, independent of w. We associate with n the name and notion of band "corrugation," although that does not lead to any more rigorous or general

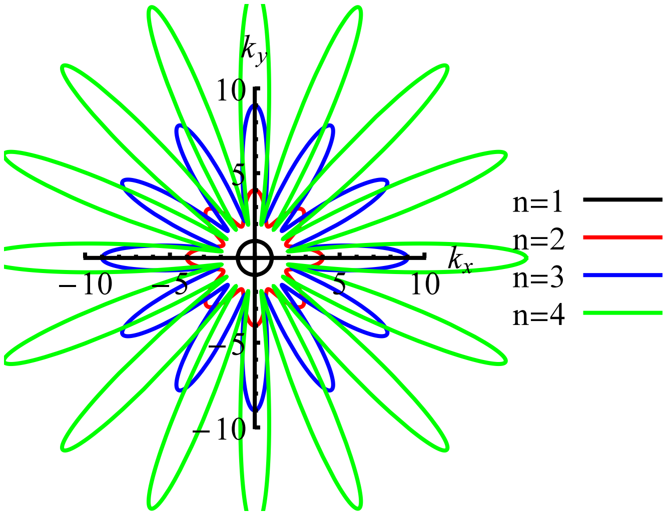

Figure 2. Angular effective mass contours of $f(\theta)$ for a two-dimensional dispersion relation of the form $E = \frac{\hbar^2 k^2}{2\mathrm{m_e}} n^2(\cos^{2n} n\theta + \sin^{2n} n\theta)$ for $n = 1$, 2, 3, and 4.

definition. Nevertheless, the basic idea of "corrugation" is that it increases with increasing number of radial "valleys." One may expect that the DOS effective mass increases correspondingly. Although that is often the case, it is not always so, as this example demonstrates. Analytic expressions for the band warping parameter, w, and the DOS effective mass, m_*, can still be derived, but they are fairly elaborate and we omit them here. Suffice it to say that w increases monotonically with n, whereas m_* at first decreases with n, but then it reaches a minimum, after which m_* increases monotonically with n. Corresponding plots of w and m_* are shown in Fig. 3. This example thus demonstrates that w and m_* do not necessarily correlate with each other, nor with the notion of band "corrugation."

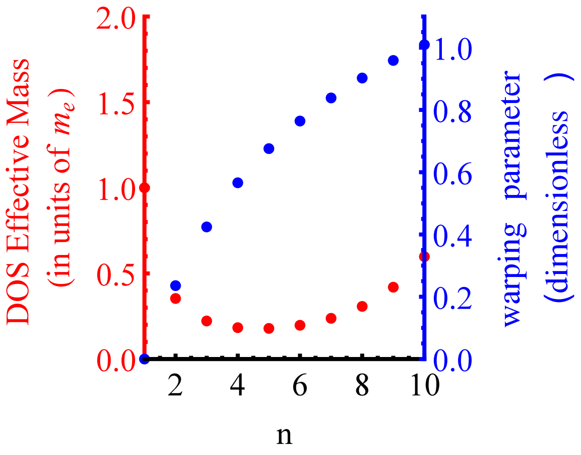

Figure 3. Warping parameter w (in blue) and the DOS effective mass m_* (in red and in units of $\mathrm{m_e}$) corresponding to Eq. (14). While w increases monotonically with n, m_* decreases at first, but subsequently increases with n.

Corrugated example with $w = 0$

We have thus demonstrated that the band warping parameter w does not necessarily increase or correlate with an increasing DOS effective mass m_*. In fact, looking back at Fig. 1 of the main paper[1], we can easily draw parametrized curves where w decreases while m_* increases. We can also draw curves in Fig. 1 of the main paper[1] where w stays constant while m_* either increases or decreases.

Let us then provide a two-dimensional example that has $w = 0$, although the energy dispersion is *not* twice-differentiable at the origin, and m_* still decreases at first, and then increases with increasing corrugation or n. Consider the energy dispersion $E = \frac{\hbar^2 k^2}{2\mathrm{m_e}} f(\theta) = \frac{\hbar^2 k^2}{2\mathrm{m_e}} \left((n^2 - 10n + 30) \left(2 + \cos 2(2n-1)\theta \right) \right)$, whose angular effective mass $f(\theta)$ is plotted in Fig. 4 for $n = 1$, 2, 3, and 4. We can prove that the band warping parameter is always $w = 0$, independently of corrugation or n, but the DOS effective mass m_* at first increases with corrugation, then it reaches a maximum at $n = 3$, and subsequently decreases monotonically for all $n > 3$.

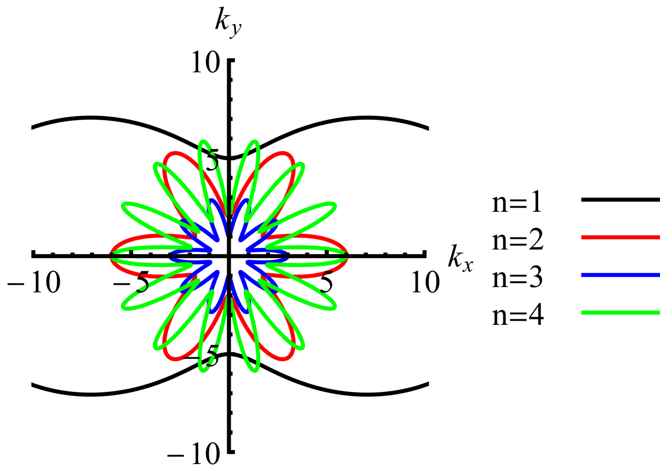

Figure 4. Angular effective mass contours of $f(\theta)$ for a two-dimensional dispersion relation of the form $E = \frac{\hbar^2 k^2}{2\mathrm{m_e}} f(\theta) = \frac{\hbar^2 k^2}{2\mathrm{m_e}} \left((n^2 - 10n + 30) \left(2 + \cos 2(2n-1)\theta \right) \right)$ for $n = 1$, 2, 3, and 4.

This example demonstrates that a function that is *not* twice-differentiable at an isolated point can still have $w = 0$. Care must then be used in drawing conclusions about band warping from use of any particular warping parameter or any notions of corrugation.

References

1. Mecholsky, N. A., Resca, L., Pegg, I. L. & Fornari, M. Density of States for Warped Energy Bands. *Sci. Rep.* **?**, ? (2015).

2. Bassani, F. & Pastori Parravicini, G. *Electronic States and Optical Properties in Solids* (Pergamon, Oxford, 1975).

3. Mecholsky, N. A., Resca, L., Pegg, I. L. & Fornari, M. Theory of band warping and its effects on thermoelectronic transport properties. *Phys. Rev. B* **89**, 155131 (2014).
